# Metabolic syndrome and carotid intima-media thickness in chronic obstructive pulmonary disease

**DOI:** 10.1186/2049-6958-8-61

**Published:** 2013-09-17

**Authors:** Aylin Ozgen Alpaydin, Isin Konyar Arslan, Selim Serter, Aysin Sakar Coskun, Pinar Celik, Fatma Taneli, Arzu Yorgancioglu

**Affiliations:** 1 Department of Pulmonary Diseases, Dokuz Eylul University Medical Faculty, Inciraltı, 35340 Izmir, Turkey; 2 Department of Pulmonary Diseases, Celal Bayar University Medical Faculty, Manisa, Turkey; 3 Department of Radiology, Celal Bayar University Medical Faculty, Manisa, Turkey; 4 Department of Biochemistry, Celal Bayar University Medical Faculty, Manisa, Turkey

**Keywords:** Atherosclerosis, Carotid IMT, COPD, Metabolic syndrome, Serum CRP

## Abstract

**Background:**

The aim of this study is to investigate the prevalence of metabolic syndrome (MetS), carotid intima media thickness (IMT), and serum C-reactive protein (CRP) levels in patients with chronic obstructive pulmonary disease (COPD), and the possible relationships among them.

**Methods:**

Fifty stable COPD patients and 40 healthy controls were included in the study. The participants were further divided into four groups according to their smoking status. Pulmonary function tests were performed in COPD patients. Anthropometric measurements and blood chemistry analysis, serum CRP levels and carotid intima-media thickness (IMT) measurements were performed in all the study population.

**Results:**

Prevalence of metabolic syndrome was 43% in COPD patients and 30% in the control group (p = 0.173). FEV_1_% and FEV_1_/FVC were higher in COPD patients with MetS (p = 0.001 and p = 0.014, respectively) compared to those without MetS. Prevalence of MetS was significantly different among the COPD patients with different stages (p = 0.017) with the highest value in stage 2 (59%). Carotid IMT was significantly higher in COPD patients than in control group (1.07 ± 0.25 mm and 0.86 ± 0.18 mm, respectively; p < 0.001). Serum CRP levels were not different in COPD patients and controls, however they were higher in individuals with MetS compared to those without MetS regardless of COPD presence (p = 0.02).

**Conclusions:**

Early markers of atherogenesis, in terms of carotid IMT, were found to be higher in COPD patients than in healthy controls. MetS prevalence was observed to decrease as the severity of airflow obstruction increased. Therefore, screening COPD patients for these cardiovascular risk factors would be a novel approach even in absence of symptoms.

## Background

Chronic obstructive pulmonary disease (COPD) is one of the leading causes of mortality and morbidity worldwide [1]. It has been regarded as a respiratory system disease for many years; however the multisystemic nature of this disease, presenting comorbidities such as malnutrition, diabetes mellitus, skeletal muscle abnormalities and cardiovascular disease (CVD), is an area of interest currently [2,3]. Whether these comorbidities are or not related to common risk factors like smoking, ageing and genetic predisposition has not been elucidated yet [4]. Reduced forced expiratory volume in 1 second (FEV_1_) has been reported to be associated with cardiovascular risk independently from the classical risk factors [5-7]. Oxidative stress and chronic hypoxia in COPD patients may contribute to the development of CVD, but the most obvious factor is thought to be the systemic inflammation [8]. Airway inflammation may induce systemic inflammation, particularly C-reactive protein (CRP) production, which is also associated with the progression of atherosclerosis [9,10]. Furthermore, atherosclerosis has been reported to be related to COPD [11] and metabolic syndrome (MetS) [12]. Carotid atherosclerosis strongly correlates with coronary atherosclerosis [13] and carotid intima-media thickness (IMT) measured by carotid doppler ultrasound is an effective, validated method for evaluating carotid atherosclerosis [14,15].

Metabolic syndrome (MetS) is defined as a cluster of components associated with excessive adiposity due to overnutrition and sedentary life style. These components are abdominal obesity, insulin resistance, dyslipidemia and increased blood pressure [16]. CVD risk is increased by 2 times [17], whereas type 2 diabetes mellitus (DM) is increased by 5 times in MetS [18]. The components of the metabolic syndrome have been observed more frequently in COPD patients respect to controls and MetS risk was reported to be increased in individuals with airflow obstruction [19,20].

In the light of the above mentioned observations, this study aims to investigate the common cardiovascular risk factors like MetS, carotid IMT - as an indicator of carotid atherosclerosis - and CRP levels and the relationships between these parameters in COPD patients. The measurement of carotid IMT and the diagnosis of MetS may enable us to use these parameters in COPD patients to predict atherosclerosis and CVD, pathological conditions that can seriously affect the outcome.

## Methods

### Study design

Between April 2009 and April 2010, 65 consecutive COPD patients, who referred to our pulmonary diseases outpatient clinic and 50 age matched healthy controls from family members of hospitalized patients, were screened for study eligibility. Out of them, 50 COPD patients and 40 healthy controls were recruited, while 25 additional candidates were excluded on the basis of the inclusion and exclusion criteria. Inclusion criteria for COPD patients were; age > 50 years and stable state (no exacerbations and no medication change in the last 6 weeks). COPD diagnosis was based on a history of smoking more than 20 pack/years and a FEV_1_/ forced vital capacity (FVC) ratio of less than 70% 20 min after salbutamol administration [1]. Inclusion criteria for the control group were: age > 50 years, absence of COPD as confirmed by history and physical and/or spirometric evaluation. Exclusion criteria for both COPD and control groups were: presence of an inflammatory comorbidity (e.g. inflammatory bowel diseases, rheumatologic diseases, vasculitis), acute infections, respiratory diseases other than COPD, history of coronary heart disease and/or decompensated cardiovascular disease and uncontrolled diabetes mellitus. The study was approved by the human-research review board of Celal Bayar University and all participants gave a written informed consent. The study population was grouped according to patients’ smoking status as follows: smoker COPD group (n = 33); non-smoker COPD group (n = 17); smoker control group (n = 21); and non-smoker control group (n = 19).

Demographic features, medical history, comorbidities and smoking status of the study population were recorded. Non-smokers were defined as never smokers or ex smokers. The patients with a history of less than 10 years of smoking or who quitted smoking at least 20 years before were accepted as ex smokers. Blood pressure, weight, height, and waist circumference measurements were performed. Venous blood samples were obtained for the analysis of serum CRP level. Bilateral carotid arterial Doppler ultrasonograpy was performed for the measurement of carotid IMT. COPD patients also underwent pulmonary function tests.

### Anthropometric measurements

The height and weight of the study population were measured in light indoor clothes and without shoes [21]. Body mass index (BMI) was calculated as described previously [22]. Waist circumference was determined by a single observer using a tapeline at the midpoint between the lowest rib and the iliac crest [23].

#### Blood pressure measurement

Blood pressure was measured by a digital sphygmomanometer (Omron M2 Compact, Omron, Japan) after 10 minutes of resting according to the American Heart Association’s recommendations [24]. The mean of the last three measurements was recorded.

### Blood biochemistry

After an overnight fasting, venous blood sample (10 ml) was obtained. Serum glucose, triglyceride and high-density lipoprotein (HDL)-cholesterol levels were measured with standard methods using a chemical analyzer (Beckman Coulter UnicelDxC 800 Synchron Clinical System, USA).

### Evaluation of metabolic syndrome

Metabolic syndrome was defined as abdominal obesity (defined as a waist circumference of 95 cm in males and 80 cm in females) plus any two of the four following criteria:

(1) increased blood pressure (130/85 mmHg);

(2) insulin resistance (fasting plasma glucose (FPG) ≥ 100 mg);

(3) increased triglyceride levels (≥ 150 mg/dl);

(4) reduced HDL–cholesterol level (< 40 mg/dl for men, < 50 mg/dl for women) according to the International Diabetes Federation (IDF) criteria [16].

Participants who already were on anti-hypertensive drugs or oral hypoglycemic agents/insulin were regarded as meeting the criteria.

### Measurement of the serum CRP level

Venous blood samples were centrifuged and the sera were stored at −20 C. Serum CRP levels were assessed using an analyzer (Immulite 2000, Diagnostic Products Corporation DPC Los Angeles, CA, USA) by solid-phase chemiluminescenceimmunometric assay method with original commercial reagents (Immulite 2000, Siemens Healthcare Diagnostics Products Limited Gwynedd, United Kingdom). Intra assay and inter assay coefficients of variation were < 8.7% at different concentrations.

### Pulmonary function tests

Pulmonary function tests were performed using Jaeger Master Screen Pneumo V452I device. FEV_1_, FVC, FEV_1_/FVC were measured according to the American Thoracic Society criteria [25]. COPD staging was done according to GOLD 2009 [1].

### Measurement of the carotid intima-media thickness

Bilateral carotid arteries were evaluated by a single trained radiologist blind to clinical evaluation using a B-mode ultrasonography (General Electric, Logic 3 Expert Ultrasound) with a 5–10 MHz multi frequency linear probe. The luminal diameters of the bilateral common carotid arteries and internal carotid arteries were measured between the bright internal layers of the parallel vessel walls. Intima**-**media thickness was defined as the distance between the edge of the luminal echo and media/adventitia layer. All subjects had IMT measurements at the proximal, middle and distal levels of both common carotid arteries. The mean thickness at these three points was calculated for each carotid artery and the highest value was accepted as IMT. Measurements with a focal IMT of 1 mm or greater were defined as increased IMT [26].

### Statistical analysis

The data were expressed as the mean and standard deviation (SD) or the median and interquartile range. The study groups were compared using an unpaired t test and one-way ANOVA for continuous variables and Mann Whitney U-test for variables with non-normal distribution. Chi-square analysis was used for the comparison of categorical data. Univariate correlation analysis between IMT, FEV_1_%, FEV_1_/FVC, serum CRP levels was performed by calculating Pearson’s or Spearman’s correlation coefficients. Furthermore, a multivariate logistic regression model was utilized to analyze the association between the presence of COPD and MetS. A multivariate linear regression model was utilized to analyze the relationship between the presence of COPD and IMT. These regression models were used to control the potential confounding factors (namely: age, BMI, smoking, hypertension, FPG, triglyceride, HDL-cholesterol). Levels of CRP were log-transformed for all analyses. All data were analyzed by SPSS 15.0 software package. p ≤ 0.05 was considered to be statistically significant.

## Results

### Characteristics of the study population

Demographic, clinical and laboratory features of the COPD patients (n = 50) and control group (n = 40) are shown in Table 1. COPD patients and control group were similar for age, gender, and concomitant diseases. The smoker COPD group and that of smoker controls were not statistically different according to age (p = 0.11).

**Table 1:** Demographic and laboratory parameters of the study population

	**COPD group**	**Control group**	**p**
	**(n = 50)**	**(n = 40)**	
**Demographic parameters**			
Age (year) (mean ± SD)	61.3 ± 6.4	58.4 ± 8.4	0.08
Gender (male/female) (n)	45/5	34/6	0.47
Smokers (n,%)	33 (66%)	21 (52%)	0.19
Concomitant disease (n,%)	12 (24%)	6 (15%)	0.29
BMI (kg/m^2^) (mean ± SD)	27.2 ± 5.0	27.6 ± 4.7	0.74
High BP (BP > 130/85 mmHg) (n,%)	22 (44%)	15 (38%)	0.53
**Laboratory parameters**			
***Pulmonary***			
FEV_1_ (%) (mean ± SD)	46.3 ± 16.8	-	-
FEV_1_ (L) (mean ± SD)	1.42 ± 0.64	-	-
FEV_1_/FVC (%) (mean ± SD)	53.0 ± 9.0	-	-
***Metabolic***			
FPG (mg/dL) (mean ± SD)	101.8 ± 23.0	93.0 ± 10.8	**0.02**
HDL-cholesterol (mg/dL) (mean ± SD)	42.7 ± 11.5	40.5 ± 9.7	0.33
Triglyceride (mg/dL) (median and IQR)	90.5 (52.0)	117.0 (84.2)	**0.05**

*BMI* Body mass index, *BP* blood pressure, *COPD* Chronic obstructive pulmonary disease, *FPG* Fasting plasma glucose, *FEV*_*1*_ Forced expiratory volume in 1 second, *FVC* Forced vital capacity, *HDL-cholesterol* High-density lipoprotein- cholesterol.

#### Metabolic syndrome

Metabolic syndrome was assessed in 22 patients (44%) in the COPD group and in 12 patients (30%) in the control group (p = 0.17) (Table 2). Comorbidities in the study population were found to have no effect on MetS prevalence.

**Table 2:** Metabolic syndrome prevalence, serum CRP levels and carotid intima-media thickness in the study population

	**COPD group**	**Control group**	***p***
	**(n = 50)**	**(n = 40)**	
MetS prevalence (n,%)	22 (44%)	12 (30%)	0.17
Serum CRP (mg/dl) (median-IQR)	2.22-5.43	1.08-3.42	0.371
Carotid IMT (mm) (mean ± SD)	1.07 ± 0.25	0.86 ± 0.18	0.001

*COPD* Chronic obstructive pulmonary disease, *CRP* C reactive protein, *IMT* Intima**-**media thickness, *MetS* Metabolic syndrome.

**Figure 1:**
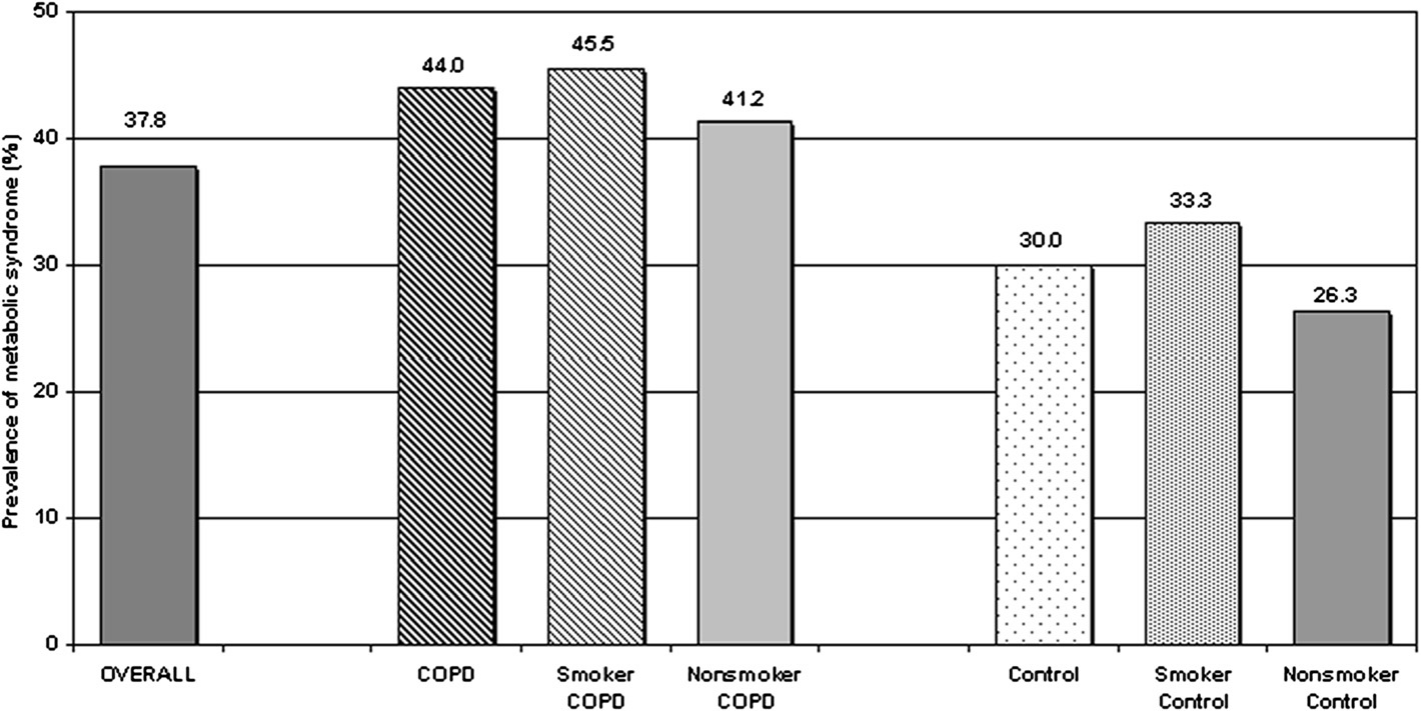
Prevalence of metabolic syndrome according to smoking status in COPD patients and controls.

**Figure 2:**
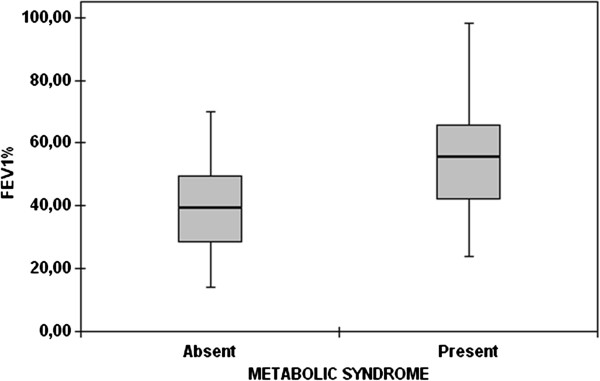
Mean forced expiratory volume in 1 second /forced vital capacity percent values of the patients with or without metabolic syndrome (the markers and the error bars denote means and 95% confidence intervals).

MetS prevalence was not significantly different among the four groups (Figure 1). The highest MetS prevalence was found in GOLD stage 2 (72%), followed by stage 1 and 3 (33% for both), and stage 4 (12%) COPD patients. When the combination of COPD stages 1 and 2 (early stages) was compared with the combination of stages 3 and 4 (late stages), MetS was found more frequent in the early stages compared to the late ones (64% vs 25%, respectively; p = 0.006). This finding was further supported by FEV_1_% and FEV_1_/FVC data (Figure 2). These parameters were higher in patients with MetS than in those without MetS (p = 0.001 for FEV_1_% and p = 0.014 for FEV_1_/FVC).

A multivariate-adjusted analysis taking into account all potential confounding factors (*i.e.* age, BMI, smoking, hypertension, FPG, triglyceride, HDL-cholesterol) revealed that the presence of MetS had no relation with COPD (beta = 1.228, p = 0.118).

### Serum C-reactive protein levels

**Figure 3:**
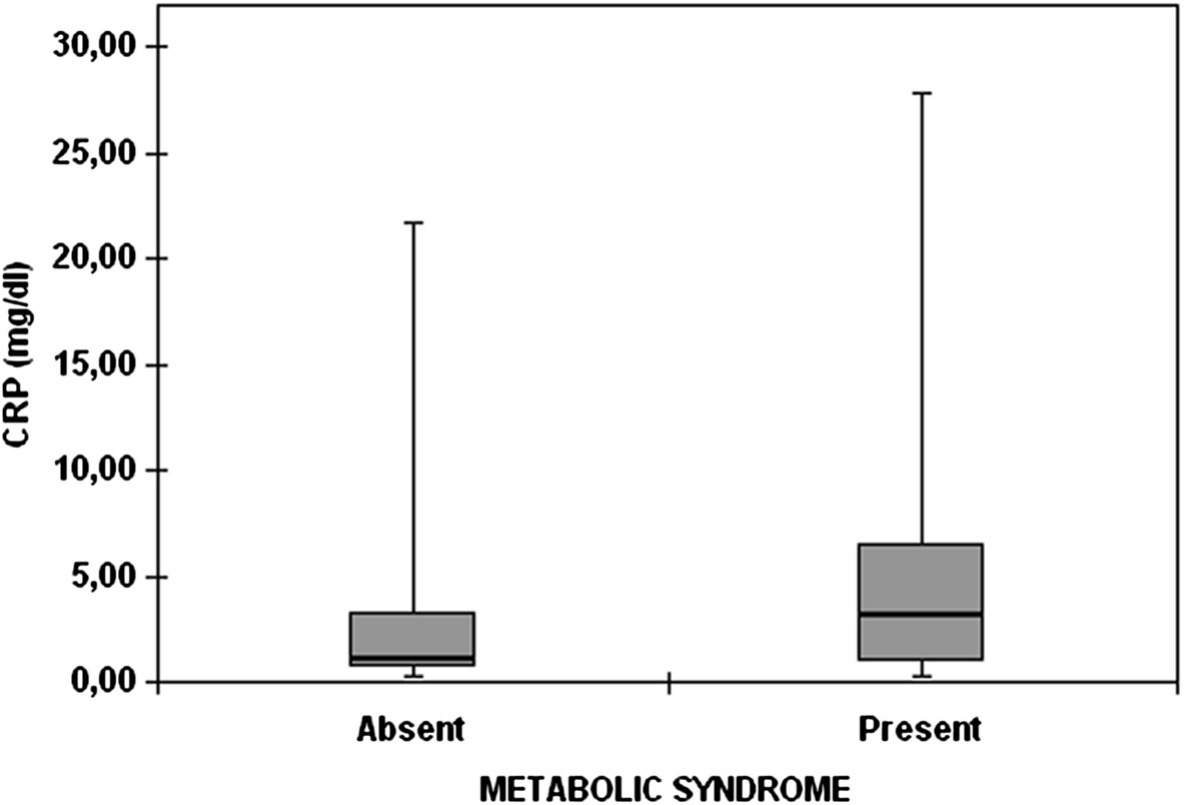
**Serum C-reactive protein levels of the patients with or without metabolic syndrome (the marker and the error bars denote median and 25**^**th**^**and 75**^**th **^**percentiles).**

There were no significant differences in serum CRP levels between COPD and control group (p = 0.371) (Table 2). Moreover, serum CRP levels did not show significant difference between COPD and control smokers (p = 0.78). It was also supported by the multivariate-adjusted analysis demonstrating no significant association between serum CRP levels and presence of COPD in all study population (beta = 0.083, p = 0.469). However, serum CRP levels were elevated in the study participants with MetS (p = 0.02) (Figure 3).

### Carotid intima-media thickness

COPD group had a statistically significant thicker carotid IM compared to controls (p < 0.001) (Table 2). Mean IMT was found to be increased in the smoker COPD group compared to smoker controls (p = 0.006). When all subjects were included in the analysis, IMT was correlated with age (r = 0.371, p < 0.001), but not with disease stage, FEV_1_% or serum CRP levels. In COPD patients with MetS, IMT did not show a significant increase compared to COPD patients without MetS (1.11 ± 0.24 mm vs. 1.04 ± 0.26 mm; p = 0.34). Another important finding was that there was no significant difference between IMT values of participants with MetS and without MetS (1.01 ± 0.25 mm vs 0.95 ± 0.24 mm; p = 0.20).

A multivariate-adjusted analysis showed that in all the study population IMT had a positive correlation with COPD (beta = 0.151, p = 0.020). Among the potential confounding factors, age and BMI were also positively correlated with IMT (beta = 0.008, p = 0.020 and beta = 0.011, p = 0.029, respectively). Other factors (*i.e.* age, smoking, hypertension, FPG, triglyceride, HDL-cholesterol) had no significant association with IMT.

A multivariate-adjusted analysis of the correlation between MetS and IMT in all study population, and in COPD patients separately, did not demonstrate a significant association.

## Discussion

COPD is known to be associated with comorbidites that account for more than 50% of the health costs related to COPD [27]. Therefore, it is important to evaluate the patients with COPD for systemic manifestations, especially for CVD. Possible explanations of the high cardiovascular morbidity and mortality observed in COPD patients are high smoking prevalence, diet and sedentary life style. Even in absence of a smoking history, FEV_1_ was reported to be correlated with cardiovascular risk [8]. Metabolic syndrome has been defined as a component of systemic inflammatory syndrome and combination of cardiovascular disease risk factors [28]. In this study we investigated the prevalence of cardiovascular comorbidities such as MetS and atherosclerosis as well as their association with systemic inflammation in a group of COPD patients. We found a relatively high MetS prevalence in COPD patients; however this was close to the prevalence observed in controls without airway obstruction. On the other hand, carotid IMT as an atherosclerosis marker was observed to be increased in COPD patients with respect to controls.

Lam et al. have measured spirometric parameters and fasting metabolic markers in 7,358 adults older than 50 years and they found a FEV_1_/FVC value at lower normal limit in 6.7% and MetS in 20% of the participants. MetS risk was determined to be greater in patients with airflow limitation than in controls when other factors were adjusted (OR = 1.47 95% CI: 1.12-1.92) [20]. Prevalence of MetS was 23.7% in general population according to NCEP: ATP III criteria, 25.1% according to World Health Organization (WHO) and 39.1% according to IDF criteria in the analyses of NHANES cohorts [29,30]. In our country, MetS prevalence was reported to be approximately 28–32.2% in men and 39.6-45% in women [31,32]. We observed the prevalence of MetS was 44% in COPD patients and 30% in the control group according to IDF criteria, however this finding was not statistically significant (p = 0.173). Considering the predominance of male population in our study, the prevalence of MetS found in the control group is compatible with the previously reported male frequency of this syndrome in our country. The high prevalence we observed in COPD patients might be highlighted by further studies, including larger number of patients, to investigate the association between MetS and airway obstruction and demonstrate a significant risk of MetS in COPD. Similarly to our results, MetS diagnosis among a small group of participants (38 COPD patients, 34 controls) attending a cardiopulmonary rehabilitation program has been reported in 47% of COPD patients and in 21% of controls and the difference was significant [19]. Watz et al. have investigated 170 COPD patients and 30 with chronic bronchitis and normal spirometry and found an overall MetS prevalence of 47.5%., while it was 53% in chronic bronchitis, and 50%, 53%, 37% and 44% in COPD stages 1, 2, 3, and 4, respectively. They have also shown high hs-CRP levels in patients with MetS [33]. In our study we found significantly different MetSprevalences in COPD patients at different stages (p = 0.017). The highest prevalence was observed in stage 2 (59%) patients, and the lowest one in those at stage 4 (4.5%), thus MetS was more frequent in the early stages of the disease (p = 0.006). However, the number of patients in the different GOLD stages was not homogeneous and most patients were at stage 2 in our study population. In addition, inour study, patients established to have MetS had higher FEV_1_% (55% vs. 40%) and CRP values (53 vs. 40 mg/dl) similarly to the study by Watz [34]. MetS is mostly diagnosed in obese patients, whereas cachexia is common in patients with advanced COPD, and this may be the reason why MetS was found more frequently in the early stages of the disease. When the participants were analyzed according to their smoking status, we found no differences in MetS frequency between COPD and control groups.

Serum CRP concentration has been proposed as a biomarker of systemic inflammation and was reported to be increased in almost all chronic diseases [34]. Besides, increased serum CRP concentration has been considered a causal factor of atherogenesis [35]. In the present study we investigated the CRP levels in COPD patients and in controls, as well as the relationships between serum CRP levels, MetS and carotid IMT in COPD patients. We were unable to see the expected difference of serum CRP levels in COPD patients compared to control group. This might be due to the small number of study subjects as well as to the similar characteristics of study and control individuals like the observed insignificant difference in MetS prevalence. Another important result of our study was that there was no correlation between serum CRP levels and carotid IMT. On the other hand, serum CRP levels were significantly higher in the study participants with MetS compared to those without MetS, regardless of COPD presence. This may suggest that systemic inflammation may be more pronounced in MetS whether it associates with COPD or not.

Mechanisms underlying the association between atherosclerosis and COPD have not been clarified yet. However, chronic systemic inflammation, hypercoagulability, platelet activation, and oxidative stress are the possible important contributors [36,37]. Studies examining the relationship between carotid atherosclerosis and lung function have reported increased IMT in patients with lower FEV_1_[38-41]. Recently, it has been reported that the decrease in FEV_1_ was independently associated with carotid atherosclerosis, and emphysema was associated with a reduced ankle-brachial index (Table 3) [42]. Similarly, in our study we observed increased carotid IMT in patients with COPD (1.07 ± 0.25 mm) compared to controls (0.86 ± 0.18) after adjusting for smoking, hypertension, fasting plasma glucose, triglyceride and HDL-cholesterol levels (p < 0.001). IMT was correlated with age (p < 0.001, r = 0.371), however there was no correlation between IMT and disease stage, FEV_1_%, or serum CRP level. When only smokers were considered, IMT was found to be higher in the COPD group compared to controls (p < 0.006). This finding might suggest that, although we could not demonstrate a direct correlation between FEV_1_% and IMT, patients with airflow obstruction are more proneto atherogenesis due to some other mechanisms beyond smoking.

**Table 3:** Studies on the relationship between airway obstruction and atherosclerosis

**Reference**	**N**	**Population**	**Design**	**Methodology**	**Comments**
Engström G, 2001 [39]	207	Smokers without history of cardiovascular disease	Cohort- cross sectional	Spirometry, calf plethysmography at 55 years, spirometry, ankle-arm blood pressure and carotid ultrasound at 68 years	The risk of developing atherosclerosis is associated with the degree of ventilatory capacity
Zureik M, 2001 [11]	656	Adults without coronary heart disease	Cohort- cross sectional	Peak expiratory flow at the baseline, carotid B-mode ultrasound at baseline and 2 and 4 years later	Reduced lung function is associated with carotid atherosclerosis in the elderly
Schroeder EB, The ARIC study, 2005 [40]	14,000	Adults	Cross-sectional	Lung function, ankle-brachial index (ABI), carotid intimal-medial thickness (IMT), presence of carotid plaques	Association between decreased FEV1 and decreased ABI/increased IMT in the full cohort
Iwamoto et al., 2009 [41]	305	Smokers with airflow limitation, age-matched control smokers, control never-smokers	Cross-sectional	Chest radiogram, spirometry, blood sampling, and carotid ultrasound	Exaggerated subclinical atherosclerosis in smokers with airflow limitation
Barr RG, et al., the MESA Lung Study, 2012 [42]	3,642	Multi-Ethnic participants aged 45–84 years without clinical cardiovascular disease.	Cross-sectional	Spirometry, carotid intima-media thickness (IMT), ankle-brachial index (ABI) and coronary artery calcium (CAC), percentage of emphysema-like lung	Association between airway obstruction and emphysema with subclinical atherosclerosis in the carotid arteries

Of course there are some limitations in our study: for instance the small size of the study population, and the distribution of the groups according to smoking status and COPD stages which were relatively heterogeneous. Another important limitation is the selection of the study population. We excluded participants with obvious ischemic heart disease and uncontrolled diabetes mellitus. This might have resulted in an underestimation of MetS prevalence in our study population, although our data are supported by previous studies. The cross-sectional design of the study is a further limitation.

## Conclusions

In conclusion, in our study we found a relatively high prevalence of MetS and subclinical atherosclerosis, measured by carotid IMT, in patients with airflow obstruction. Thus, we suggest that middle aged men who are at risk of COPD should undergo a comprehensive investigation to diagnose other possible components of systemic inflammatory syndrome beyond COPD. Furthermore, the comorbidities associated with COPD should be evaluated in every patient.

## Competing interest

The authors declare that they have not competing interest.
